# Supplementary material for: Quantitative, super-resolution localization of small RNAs with sRNA-PAINT
Source: Nucleic Acids Res. 2020 Jul 27;48(16):e96. doi: 10.1093/nar/gkaa623 (PMC7498346; doi:10.1093/nar/gkaa623)
Supplement: gkaa623_Supplemental_Files [file gkaa623_supplemental_files.zip › Supplementary Figure Legends.docx]

# Supplementary Figure Legends

## Supplementary Figure 1. sRNA-PAINT showed limited to no background with a scrambled control probe.

(**A**) sRNA-PAINT with probes for a 24-nt phasiRNA and a scrambled control. Both docking strands were detected with corresponding imager strands. Arrows indicate 2 out of ~66 fiducials used in experiments. Scrambled control detected little or no signal. Scale bar = 10 µm for all images. (**B**) qPAINT analysis of background level of LNA probes with scrambled controls. Number of the binding events for each calculated area (20 pixel size diameter circle in Picasso software) in different cell layers were plotted. A mean of 5.12 binding sites were calculated and used as background level for all subsequent calculations. Ten locations in each cell layer were picked for qPAINT quantification. Three replicates and a total of 150 samples were used for the background calculation (n=150).

## Supplementary Figure 2. Quantification of specificity of VARNISH probes.

VARNISH 24-nt phasiRNA probe (V), a probe with 2-nt mutations (m2), and probe no LNAs (nL) were applied to each anther sample. Quantification was done using qPAINT analysis. Numbers show the binding events for each cell in different cell layers. An average of 95% reduction is detected with m2 in all cell layers. 10 different locations in each specified cell layer and three replicates (two replicates for nL) were taken for statistical analysis (n=150 for V and m2, n=100 for nL). A no LNA probe with 2 nt mutation was used as control for nL probe for qPAINT calculation. Letters indicate significant differences using Tukey’s test (P < 0.05).

## Supplementary Figure 3. VARNISH probe signals remain stable during imaging.

Number of localization spots detected during the imaging process of 20,000 frames remain relatively stable for 24-nt phasiRNA.

## Supplementary Figure 4. sRNA-Exchange-PAINT for miR2275 and 24-nt phasiRNA co-localization.

(**A**) A diagram showing sRNA-Exchange-PAINT, which differs from sRNA-PAINT via the use of multiple imager strands. Imager strand 1 is first introduced to the sample, and after imaging, imager strand 1 is removed with a buffer wash. Next, imager strand 2 is introduced. This process can be repeated until the needed number of targets is reached. (**B**) sRNA-Exchange-PAINT on miR2275 (yellow) and a 24-nt phasiRNA (blue) in maize anther. After the buffer wash, imaging showed little or no signal following each round of detection using the imager strands. Scale bar = 10 µm for all images. (**C**) Colocalization with clustering analysis was conducted using Clus-DoC. Left panels show either sequential imaging of 24-nt phasiRNA and miR2275 or 24-nt phasiRNA imaged and then re-imaged. Middle panel showed the Doc heat map for each area of interest. A Doc score close to 1 indicates perfect colocalization, 0 indicates random distribution, and -1 indicates segregation. Right panels show a frequency map of Doc scores. 24-nt phasiRNA and miR2275 showed localization rates of around 29.8% and 27.2% for each channel. 24-nt phasiRNA imaged and then re-imaged showed localization rates of around 68.7% and 64.3% for each channel.

## Supplementary Figure 5. Multiplexed detection of phasiRNA biogenesis components in different imager strand order.

Quantification of the five RNA candidates in pollen mother cell area using different sequential orders of imager strands. No significant difference was detected. T-tests were used for statistical analysis (n=5).

# Supplementary Tables

*Supplementary Table 1. Information for sequential image of phasiRNA generation components using sRNA-Exchange-PAINT*.

*Supplementary Table 2. Probes for smFISH of a* PHAS *precursor.*
