# Supplementary figures and images for: Quantitative, super-resolution localization of small RNAs with sRNA-PAINT
Source: Nucleic Acids Res. 2020 Jul 27;48(16):e96. doi: 10.1093/nar/gkaa623 (PMC7498346; doi:10.1093/nar/gkaa623)

Supplementary Fig. 1

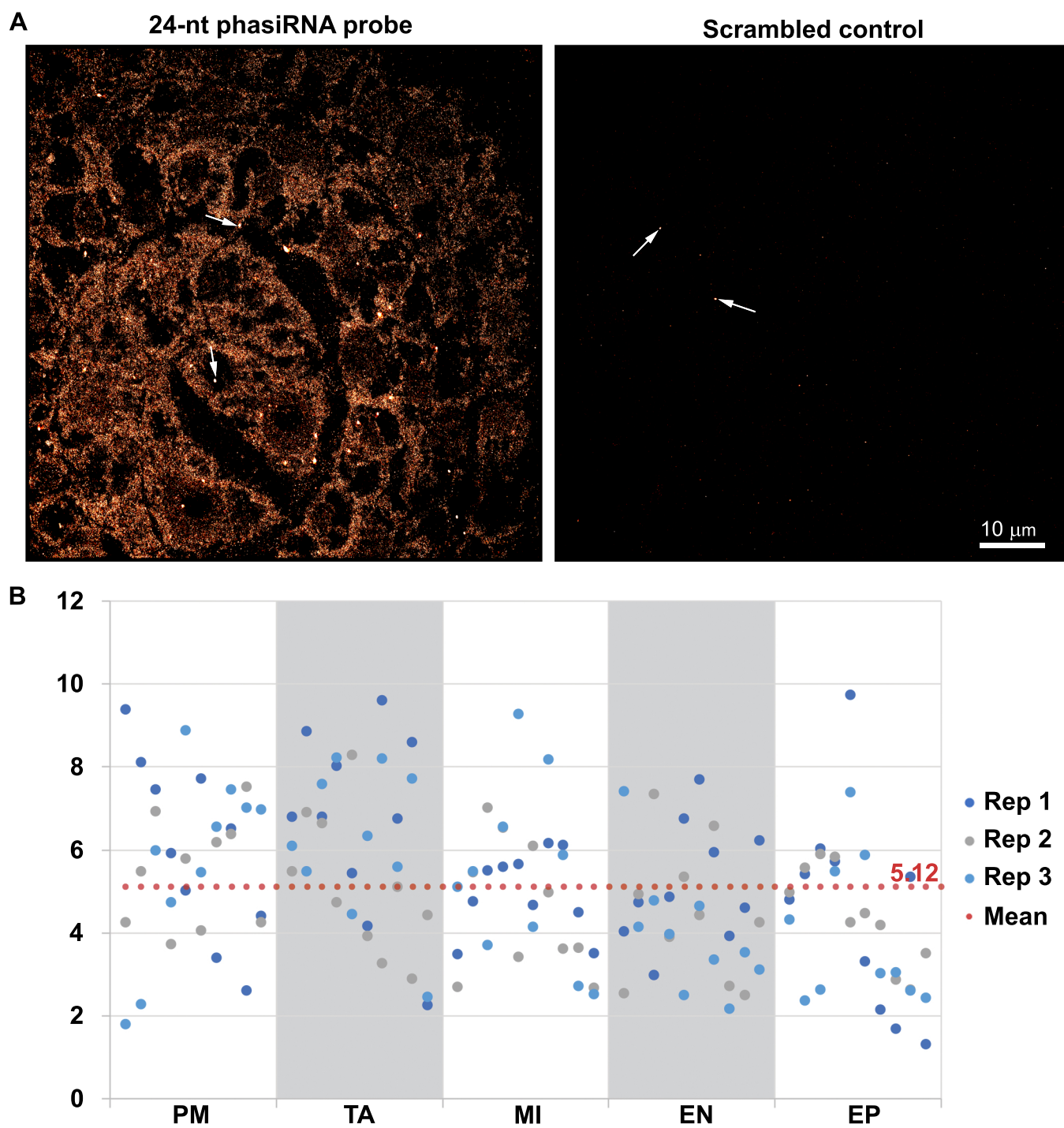

Supplement: gkaa623_Supplemental_Files [file gkaa623_supplemental_files.zip › Supplementary Figure 1_r.pdf]

Supplementary Fig. 2

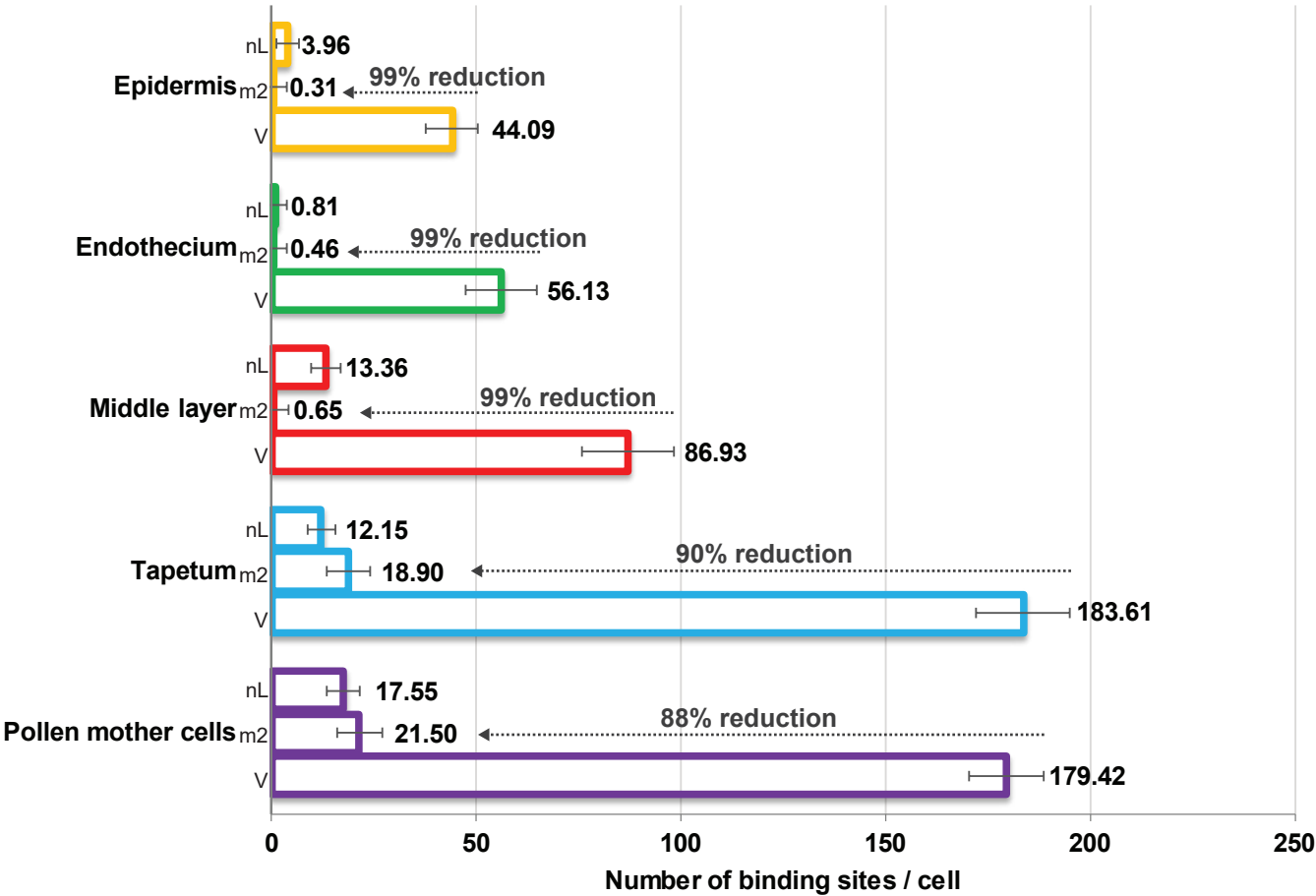

Supplement: gkaa623_Supplemental_Files [file gkaa623_supplemental_files.zip › Supplementary Figure 2_r.pdf]

**Supplementary Fig. 3**

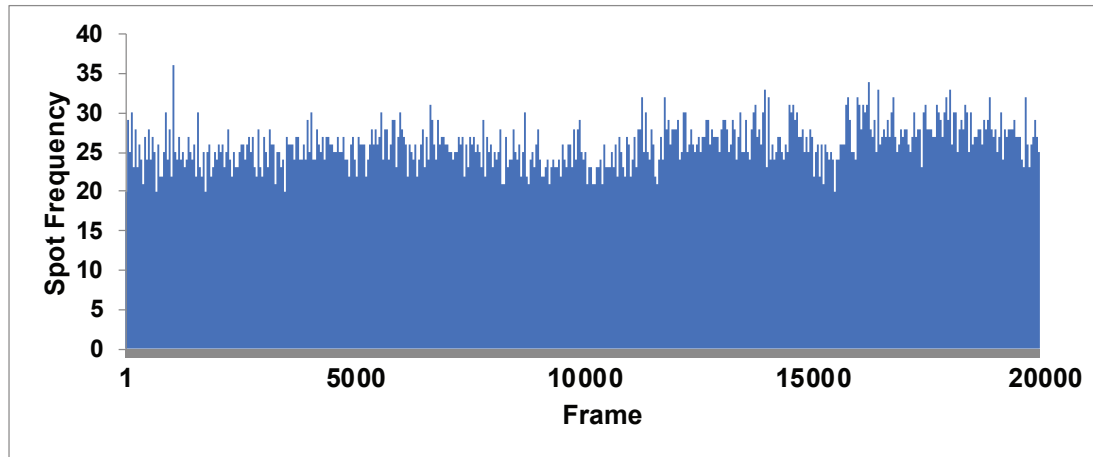

Supplement: gkaa623_Supplemental_Files [file gkaa623_supplemental_files.zip › Supplementary Figure 3_ra_show1.pdf]

Supplementary Fig. 4

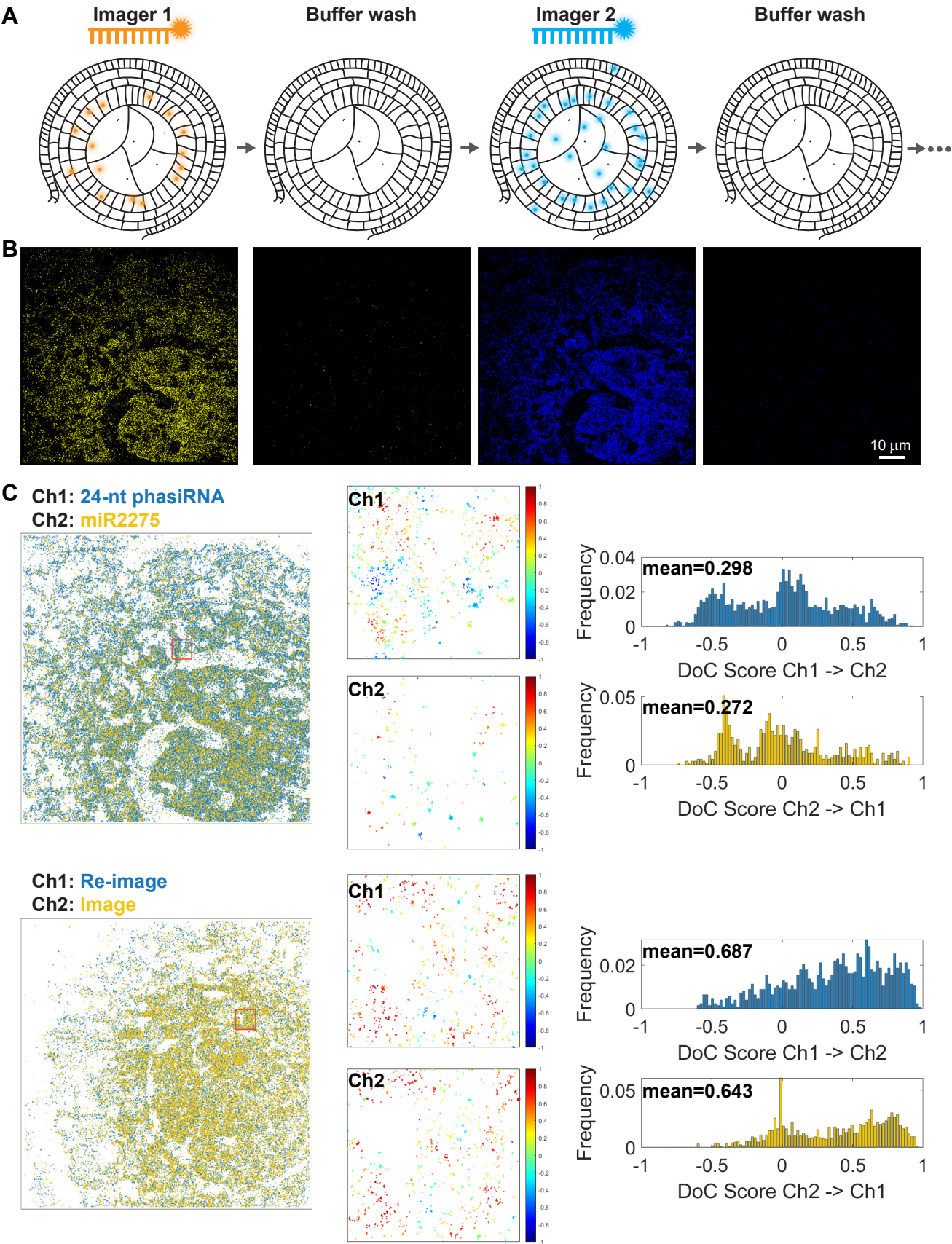

Supplement: gkaa623_Supplemental_Files [file gkaa623_supplemental_files.zip › Supplementary Figure 4_r2_v3.pdf]

Supplementary Fig. 5

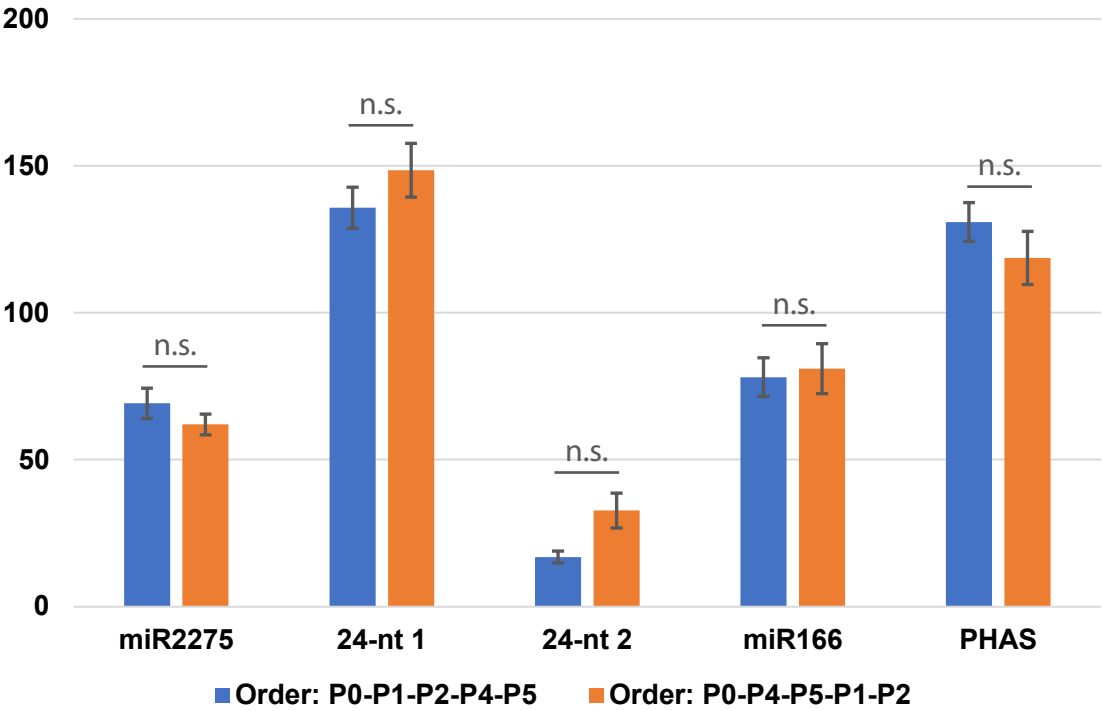

Supplement: gkaa623_Supplemental_Files [file gkaa623_supplemental_files.zip › Supplementary Figure 5_r2_v1.pdf]
